# Frequent concerted genetic mechanisms disrupt multiple components of the NRF2 inhibitor KEAP1/CUL3/RBX1 E3-ubiquitin ligase complex in thyroid cancer

**DOI:** 10.1186/1476-4598-12-124

**Published:** 2013-10-20

**Authors:** Victor D Martinez, Emily A Vucic, Larissa A Pikor, Kelsie L Thu, Roland Hubaux, Wan L Lam

**Affiliations:** 1 BC Cancer Research Centre, BC Cancer Agency, 675 West 10th Avenue, Vancouver, BC V5Z1L3, Canada

**Keywords:** KEAP1/CUL3/RBX1 E3-ubiquitin ligase complex, Gene disruption, NRF2, Thyroid cancer

## Abstract

**Background:**

Reactive oxygen species contribute to normal thyroid function. The NRF2 oxidative response pathway is frequently and constitutively activated in multiple tumor types, including papillary thyroid carcinoma (PTC). Genetic mechanisms underlying NRF2 pathway activation in PTC are not fully understood. Thus, we aimed to determine whether inactivating patterns of DNA-level alterations affect genes encoding for individual NRF2 inhibitor complex components (CUL3/KEAP1/RBX1) occur in PTC.

**Findings:**

Combined patterns of epi/genetic alterations for KEAP1/CUL3/RBX1 E3 ubiquitin-ligase complex components were simultaneously interrogated for a panel of 310 PTC cases and 40 adjacent non-malignant tissues. Data were obtained from The Cancer Genome Atlas project. Enrichment of NRF2 pathway activation was assessed by gene-set enrichment analysis using transcriptome data. Our analyses revealed that PTC sustain a strikingly high frequency (80.6%) of disruption to multiple component genes of the NRF2 inhibitor complex. Hypermethylation is the predominant inactivating mechanism primarily affecting KEAP1 (70.6%) and CUL3 (20%), while copy number loss mostly affects RBX1 (16.8%). Concordantly, NRF2-associated gene expression signatures are positively and significantly enriched in PTC.

**Conclusions:**

The KEAP1/CUL3/RBX1 E3-ubiquitin ligase complex is almost ubiquitously affected by multiple DNA-level mechanisms and downstream NRF2 pathway targets are activated in PTC. Given the importance of this pathway to normal thyroid function as well as to cancer; targeted inhibition of NRF2 regulators may impact strategies for therapeutic intervention involving this pathway.

## Introduction

**Figure 1:**
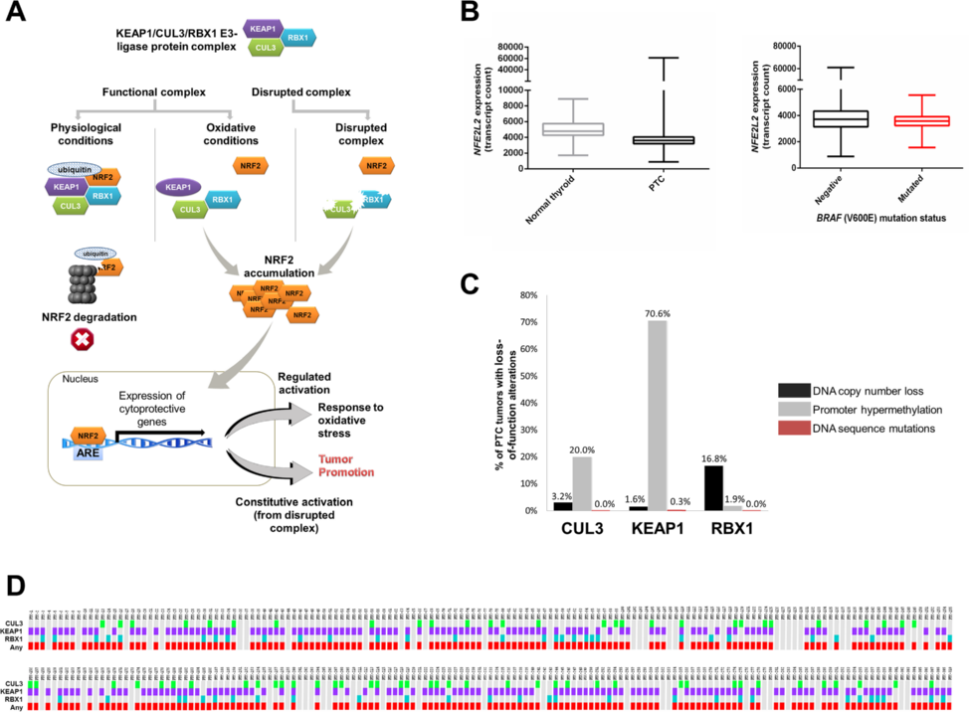
**KEAP1/CUL3/RBX1 E3-ligase protein complex. (A)** KEAP1/CUL3/RBX1 E3-ligase protein complex exert different functions under physiological and oxidative conditions. In the absence of oxidative stress (left path), NRF2 protein level and activity is maintained at low levels through interaction with the KEAP1/CUL3/RBX1 E3-ubiquitin ligase complex. When NRF2 interacts with KEAP1, it is targeted for ubiquitination and proteasomal degradation. Under oxidative conditions (middle path), KEAP1 undergoes conformational changes, which impedes interaction with NRF2, resulting in its accumulation and translocation to the nucleus. When the KEAP1/CUL3/RBX1 E3-ubiquitin ligase complex is disrupted by absence or alteration of any of its components (right path), NRF2 is stabilized, accumulated and translocate to the nucleus. In the nucleus, NRF2 can induce expression of genes containing NRF2-regulatory sequence motifs (e.g., antioxidant response elements, AREs). Constitutive activation of NRF2, as a result of complex disruption, has been linked to tumor promotion. **(B)** On the left: expression levels of *NFE2L2* gene in normal thyroid (grey) and tumors (black). Levels of *NFE2L2* were significantly higher (p < 0.0001, Mann Whitney test) in tumors compared with normal tissue. On the right: Comparison of mRNA expression levels between samples carrying *BRAF* V600E mutation (red) versus those without alteration (black) **(C)** Different types of DNA level alterations affecting each individual complex. Figure shows the patterns and frequency of DNA copy number losses (black), hypermethylation (grey), and mutation (red) affecting each complex component **(D)** Status of individual complex components across a panel of 310 PTC tumors. Each row represents an individual complex component affected by any disrupting mechanisms: CUL3 (green), KEAP1 (purple), and/or RBX1 (blue). Presence of any alteration on an individual complex component is showed in red.

Production of reactive oxidative species (ROS) by thyroid cells is essential to normal hormonogenesis and growth of the thyroid gland [1,2]. However, increased production of ROS is damaging to cells, and therefore pathways controlling cellular defensive mechanisms in response to ROS, such as the NFE2-related factor 2 (NRF2) pathway are of particular importance to the thyroid. Paradoxically, constitutive activation of this pathway occurs in several tumor types affecting multiple oncogenic functions [3] (Figure 1A). Recently, the NRF2 pathway was found activated in papillary thyroid carcinoma (PTC) [4].

Activating mutations in the NRF2 gene, *NFE2L2*, are not common in PTC and genetic mechanisms underlying NRF2 activation remain to be elucidated for this tumor type [4]. NRF2 protein levels are negatively regulated by the KEAP1/CUL3/RBX1 E3 ubiquitin-ligase complex [5] (Figure 1A). Disruption of any complex component is sufficient to disrupt complex function and activate NRF2, highlighting the importance of simultaneously examining alterations to multiple complex components [6].

Since tumor genomes are disrupted by multiple genetic mechanisms, integration of multi-‘omics data for individual tumors represents a powerful approach for discovering genes frequently altered. Therefore, we simultaneously investigated DNA sequence mutations, gene DNA copy number, and DNA methylation affecting key components of the KEAP1/CUL3/RBX1 complex and the *NFE2L2* gene using a cohort of 310 PTC tumors from The Cancer Genome Atlas (TCGA) project (Additional file 1). We hypothesize that component gene disruption (DNA level) of the KEAP1/CUL3/RBX1 complex is a frequent event in PTC and may explain activation of NRF2 previously observed in PTC. We report a remarkably high frequency of DNA disruption to NRF2 inhibitor complex components and gene expression patterns in PTC tumors concordant with NRF2 activation.

### Analysis of mutations affecting NRF2 activation

We first examined whether known mutational *NFE2L2* events were frequent in the PTC cohort. No *NFE2L2* mutations were detected, and only 1 out of 310 tumors (0.32%) exhibited *NFE2L2* copy number gains. NRF2 protein overexpression has recently been described in PTC; however, protein information was not available for this cohort. Therefore, we assessed *NFE2L2* mRNA expression in PTC tumors compared to non-malignant tissues, and found that underexpression of *NFE2L2* in tumors reached statistical significance (p < 0.0001) when compared to normal tissue (Figure 1B). Additionally, we examined 6,244 unique thyroid cancer tumors in the Catalogue of Somatic Mutations in Cancer (COSMIC). Of these, 128 (2.05%) harbor KRAS mutations. Since regulation of NRF2 normally occurs through protein degradation, this mRNA finding was not surprising.

We were also interested in evaluating *NFE2L2* mRNA expression in relation to *BRAF* and *KRAS* mutations, since mutations to these genes occur frequently in PTC [7], and mutated *KRAS* and *BRAF* are associated with increased transcription of *NFE2L2* in other cancers [8]. Three PTC tumors (<1%) exhibited mutations in *KRAS* (affecting the Ras domain) whereas 177 (57.1%) sustained *BRAF* mutations (predominantly V600E). Differential *NFE2L2* mRNA expression between tumors with and without *BRAF* mutations was not evident (Figure 1B). Taken together, NRF2 pathway activation in PTC likely occurs through molecular mechanisms independent of those affecting *NFE2L2, BRAF* or *KRAS*.

### KEAP1/CUL3/RBX1 complex is frequently disrupted in PTC

We next investigated inactivating DNA alterations affecting genes encoding the NRF2 inhibitor E3-ubiquitin ligase complex, specifically: *KEAP1*, *CUL3* and *RBX1*. We calculated the frequency of copy number loss (CNL), promoter hypermethylation and/or mutations affecting these genes. Overall, frequencies of disruption were extraordinarily high and also varied amongst genes (KEAP1, 71.2%; CUL3, 21.9%; RBX1, 18.4%). Hypermethylation was the main mechanism affecting *KEAP1* (70.6%) and *CUL3* (20%), while *RBX1* was almost exclusively affected by CNL (16.8%) (Figure 1C, Additional file 2). Sequence mutations were not identified except for one *KEAP1* G477S mutation (Figure 1C). Strikingly, when complex disruption was considered cumulatively, 80.6% of PTCs harbored a DNA alteration in at least one of the complex components (Figure 1D).

These findings indicate that multiple DNA mechanisms simultaneously affect different components of the KEAP1/CUL3/RBX1 complex at a very high frequency in PTC. Moreover, these results provide a plausible DNA level mechanism for recent findings by others describing NRF2 pathway activation in PTC [4].

### Multiple NRF2-related functions are significantly enriched among aberrantly expressed genes in PTC

**Figure 2:**
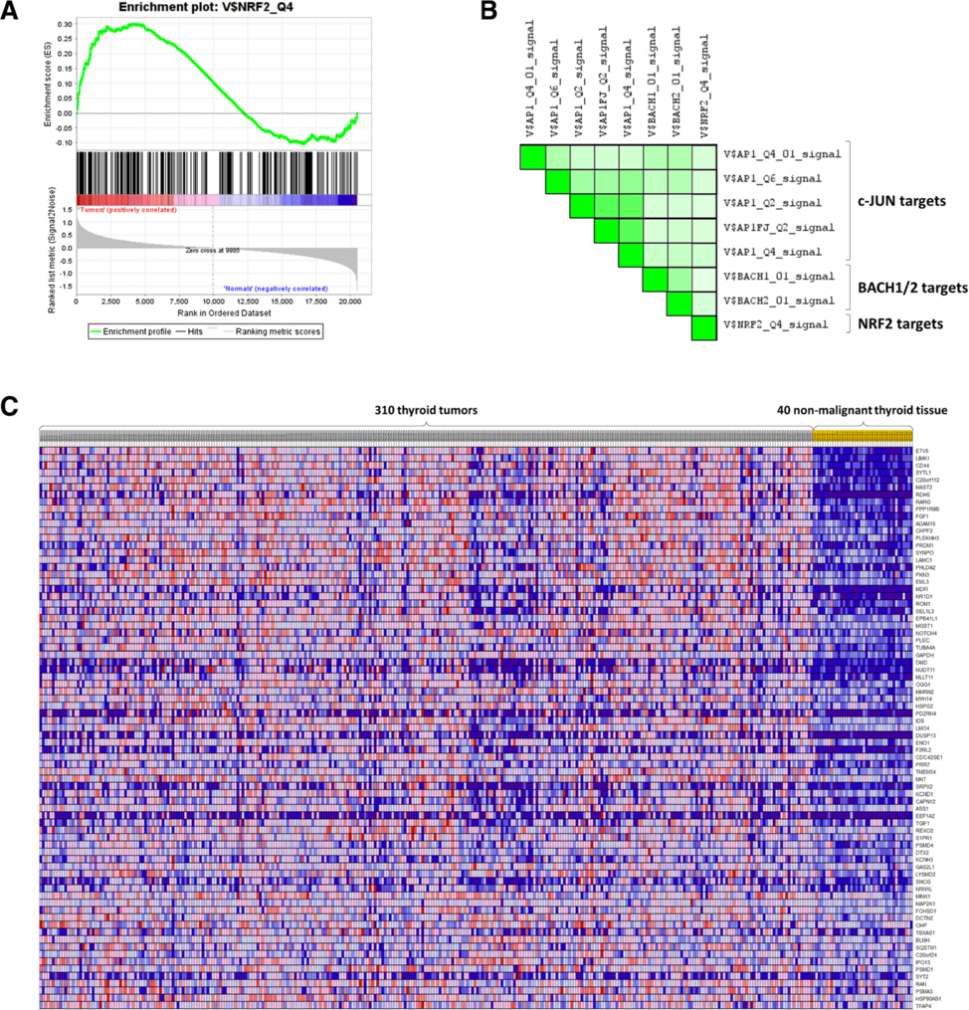
**PTC tumors are significantly and positively enriched for gene sets corresponding NRF2 related functions. (A)** A gene set (V$NRF2_Q4) consisting of *NFE2L2* transcriptional targets was significantly and positively enriched in PTC tumor relative to non-malignant tissues. For PTC tumors, normalized enrichment score (ES) = 1.42, p-value = 0.023. ES (green line) represent the probability that the gene set is positively (left side) or negatively (right side) enriched in a ranked gene list. Genes are first ranked based on differences between tumor and normal groups. Ranked genes that appear in the defined gene set are indicated as “Hits” below the Enrichment profile (black hash marks). Genes ranked near the top of the gene list are underscored with a red bar, whereas genes near the bottom of the list are underscored with a blue bar. **(B)** A Leading Edge Analysis comparing whether highly ranked genes corresponding to different gene sets overlapped. Highest ranked genes refer to those most highly differentially expressed genes between tumors compared to normals. Leading edge genes (i.e. those most different at level of expression between PTC tumors and normals) that correspond to highly statistically significantly enriched gene sets overlap with NRF2 transcriptional targets. These gene sets corresponded to functions related to NRF2, such as *c-JUN* (AP1 gene sets), a well-known transcriptional activator of *NFE2L2*, and *BACH1* and *2*, which are transcriptional repressors that compete with NRF2 to bind ARE. 100% and 0% overlap in leading edge genes are indicated by dark green and white, respectively. **(C)** A gene expression heatmap was generated for NRF2 transcriptional target genes that are over-expressed in PTC tumors relative to normals. Expression values for each gene are represented as colors, where the range of colors (red, pink, light blue, dark blue) shows the range of expression values (high, moderate, low, lowest).

We next evaluated whether disruption to the NRF2 inhibitory complex in PTC was associated with increased transcription of NRF2 transcriptional targets (i.e. those containing the consensus NRF2-binding motif). Indeed, NRF2 target genes were significantly and positively enriched (nominal p-value = 0.023) in tumors relative to non-malignant thyroid tissues (Figure 2A).

Intriguingly, multiple gene sets associated with activated NRF2 were by far the most significantly enriched overall (FDR < 0.25) (Figure 2B). Notably, five independent gene sets corresponded to the well-known NRF2 activator, c-JUN [9] and two to the transcription factors BACH1 and BACH2. These findings are highly concordant with NRF2 pathway activation in PTC. Ranked genes corresponding to NRF2 transcriptional targets are presented in Figure 2C. The highest of these, ETV5, contains a consensus NRF2-binding motif and is also a target of thyroid-specific transcription factor FOXE1 [10]. FOXE1 was not overexpressed at the mRNA level in the PTC dataset (fold change = 0.77).

## Discussion

Abundance of physiological ROS results in oxidative stress which is damaging to cells and can lead to malignant transformation. Paradoxically, the NRF2 pathway, which is responsible for protecting cells against ROS damage, is constitutively activated in multiple cancer types and associated with many cancer hallmarks [11,12]. Over-activation of this pathway has recently been described in PTC [4]. Given recent findings and the importance of ROS to normal thyroid hormonogensis, elucidation of the molecular mechanisms underlying NRF2 deregulation in PTC is relevant. Here, we provide evidence of DNA mechanisms that likely promote stabilization and activation of NRF2 in PTC.

We answered questions pertaining to NRF2 activation utilizing a large panel of PTCs with multi-‘omics data from the TCGA data portal. Consistent with previous reports, activating mutations or copy number gains/amplifications targeting *NFE2L2* are uncommon in PTC [4]. Moreover, while *BRAF* V600E mutations were common, these were not associated with increased NRF2 expression in PTC, as described for other cancers [8], indicating that NRF2 pathway activation in PTC likely occurs through other mechanisms (Figure 1B).

The KEAP1/CUL3/RBX1 complex is a key negative regulator of NRF2. We applied a multi’omics approach to interrogate DNA disruptions to KEAP1/CUL3/RBX1 complex components (Figure 1A). In a previous study, Ziros *et al.*, found no correlation between KEAP1 and NRF2 protein expression levels, arguing against decreased KEAP1 levels as mechanisms for NRF2 activation [4]. Based on our results, alterations affecting other complex components, such as CUL3 and RBX1, might be also contributing to increased expression of NRF2 observed in PTC. We found that DNA copy number loss and promoter hypermethylation frequently affect individual components, that when considered together affect a remarkably high proportion of PTCs (80%). This phenomenon has been observed by our group and others in lung cancer [6,13]. However, the frequency by which multiple complex components were simultaneously disrupted, and the molecular mechanisms affecting individual components, were strikingly different for PTC (Figure 1C-D), compared to other tumor types.

Promoter hypermethylation is the predominant mechanism affecting *KEAP1* and *CUL3* in PTC. Silencing of *KEAP1* by hypermethylation has been described in lung cancer [14], and associated with stabilized NRF2 and increased expression of NRF2 target genes in colorectal cancer [15]. While *KEAP1* mutations are frequent in other cancers, they are not in PTC. However, germ-line *KEAP1* mutations have been identified in a family with multinodular goiter [16], alluding to the importance of this gene to normal thyroid physiology. Interestingly, segmental DNA losses is almost the exclusively the type of alteration affecting *RBX1.* This phenomenon has been also observed at a similar frequency in lung tumors, with a concurrent complex function disruption [6].

Gene set enrichment analysis (GSEA) revealed that not only were genes containing the consensus NRF2-binding motif over-expressed in PTCs, but overall, the most significantly enriched gene sets corresponded to functions related to NRF2, including the NRF2 activator c-JUN and the NRF2 target competitive binders, BACH1 and 2 (Figure 2B). Bach1, competes with NRF2 protein for binding to target genes, leading to inhibited expression of NQO1 and HO-1 for example [17-19]. These results indicate that in addition to DNA disruption to NRF2 complex inhibitors, the NRF2 pathway is also likely activated by increased activity of NRF2 activators and decreased activity of NRF2 gene target transcriptional repressors in PTCs.

ETV5 was the putative NRF2 transcriptional target most highly upregulated in tumors compared to controls. ETV5 is an oncogenic member of the ETS transcription factor family, associated with cell proliferation and metastasis in various cancers [20], and a known target of thyroid-specific transcription factor FOXE1. Although we did not detect overexpression of FOXE1 in PTCs at the mRNA level, further exploration of ETV5 in the context of NRF2 signaling is warranted.

Taken together, we found that i) DNA level disruption of NRF2 inhibitory complex components occurs at an extremely high frequency in PTC, ii) primarily by promoter hypermethylation, iii) NRF2 transcriptional targets are overexpressed in PTC, and iv) activation of NRF2 in PTC is complex and likely involves activation and inhibition of multiple NRF2 activators and repressors.

A major caveat of this study is the lack of protein assessment. However, the data presented here provide rationale to interrogate the role of the NRF2 pathway in PTC at the protein level, and elucidate the target molecules driving PTC phenotypes. In summary, we present compelling evidence that activation of the NRF2 pathway is extraordinarily, selectively activated in PTC and provide further rationale for exploration of this pathway as a therapeutic target in PTC.

## Competing interests

The authors have no competing interests to declare.

## Authors’ contributions

VDM and EAV contributed equally in study design, data analysis and manuscript writing. LAP, RH, KLT, WLL contributed to data interpretation and manuscript revision. All authors read and approved the final manuscript.

## Supplementary Material

Additional file 1 Supplementary Methods. Click here for file

Additional file 2: Figure S1 DNA copy number alterations affecting components of the KEAP1/CUL3/RBX1 complex. DNA copy number affecting KEAP1/CUL3/RBX1 complex components in the TCGA PTC set. Copy number is estimated based on probe intensity derived from the Genome-Wide Human SNP Array 6.0 (Affymetrix). Around 17% of samples exhibit copy number losses (in blue, see Figure legend) affecting RBX1 gene. This appears to be a particular mechanism for gene disruption affecting this complex component, since the rest of complex components exhibit alterations at copy number level in a considerably lower frequency of samples. Click here for file
