# Supplementary material for: Frequent concerted genetic mechanisms disrupt multiple components of the NRF2 inhibitor KEAP1/CUL3/RBX1 E3-ubiquitin ligase complex in thyroid cancer
Source: Mol Cancer. 2013 Oct 20;12:124. doi: 10.1186/1476-4598-12-124 (PMC4016213; doi:10.1186/1476-4598-12-124)
Supplement: Additional file 1 — Supplementary Methods. [file 1476-4598-12-124-S1.docx]

**Additional File 1**

**METHODS**

**Papillary thyroid carcinoma (PTC) multi-'omics data**

Clinical and genomic profiles for 310 PTC were obtained from The Cancer Genome Atlas Project (TCGA). Data for genome-wide gene dosage alterations (Affymetrix SNP6.0 platform), methylation (Illumina Infinium HM450 BeadChips) and mutations (exon sequencing) were processed as described [1]. Multidimensional analysis was performed on level 3 data, using TCGA criteria for calling hypermethylation (difference in beta value Tumor vs Normal > 0.1), copy number loss and mutation, and by querying the cBio Cancer Genomics Portal [2]. For methylation analysis, an averaged beta value for normal adjacent tissue was used, due to the lack of matched tissue for all samples. Analyses were focused on the following CpG sites: CUL3: cg12698349 (chr2: 225,449,008) / cg09509863 (chr2: 225,450,859); KEAP1: cg10505024 (chr19: 10,602,877) / cg20226327 (chr19: 10,602,960) / cg22779878 (chr19: 10,600,446) / cg25801292 (chr19: 10,614,272); RBX1: cg07288693 (chr22:41,348,222) / cg21454656 (chr22:41,347,267). Similarly, normalized RNA sequencing data (Illumina HiSeq 2000) data was obtained for 310 tumors and 40 adjacent non-malignant tissues and analyzed as previously described [3, 4].

**Statistical analysis**

Differences between tumor and non-malignant groups were evaluated in GraphPad software v6 using a Mann-Whitney test. Gene-set enrichment analysis (GSEA) was performed using whole transcriptome normalized mRNA levels (n= 20,074 genes) from tumor (n= 310) and adjacent non-malignant tissues (n= 40) profiles. Here, GSEA was applied to calculate the probability that a transcriptional target gene set from the Molecular Signatures Database v4.0 (Broad Institute) is significantly enriched in tumors relative to non-malignant control tissues. Two GSEA were performed using default parameters. One, against a transcription factor target motif set comprised of NFE2L2 transcriptional target genes (n=255 genes), defined as those with an NFE2L2 predicted binding motif (NTGCTGAGTCAKN) in promoter regions around [-2kb,2kb] a corresponding transcription start site (V$NRF2_Q4, MSigDB database v4.0, Broad Institute), and another against all available transcriptional target gene sets in the MSigDB database.

**References for Additional File 1:**

1. The Cancer Genome Atlas Pilot Project (TCGA). The results published here are based upon data generated by The Cancer Genome Atlas Project established by the NCI and NHGRI. TCGA 2013, <http://cancergenome.nih.gov/> .

2. Cerami E, Gao J, Dogrusoz U, Gross BE, Sumer SO, Aksoy BA, Jacobsen A, Byrne CJ, Heuer ML, Larsson E, et al: The cBio cancer genomics portal: an open platform for exploring multidimensional cancer genomics data. Cancer discovery 2012, 2:401-404.

3. Wang K, Singh D, Zeng Z, Coleman SJ, Huang Y, Savich GL, He X, Mieczkowski P, Grimm SA, Perou CM, et al: MapSplice: accurate mapping of RNA-seq reads for splice junction discovery. Nucleic acids research 2010, 38:e178.

4. Li B, Ruotti V, Stewart RM, Thomson JA, Dewey CN: RNA-Seq gene expression estimation with read mapping uncertainty. Bioinformatics 2010, 26:493-500.

Histogram of beta values for normal (green, N=45) and tumoral (red, N=310) tissues across the set of analyzed samples. The percentage of samples with beta values (y-axis) falling in a given bin interval (x-axis) is plotted. Examples of CpG cluster considered for analysis are shown for each gene: A) KEAP1 B) CUL3 C) RBX1
