# Supplementary figures and images for: Frequent concerted genetic mechanisms disrupt multiple components of the NRF2 inhibitor KEAP1/CUL3/RBX1 E3-ubiquitin ligase complex in thyroid cancer
Source: Mol Cancer. 2013 Oct 20;12:124. doi: 10.1186/1476-4598-12-124 (PMC4016213; doi:10.1186/1476-4598-12-124)

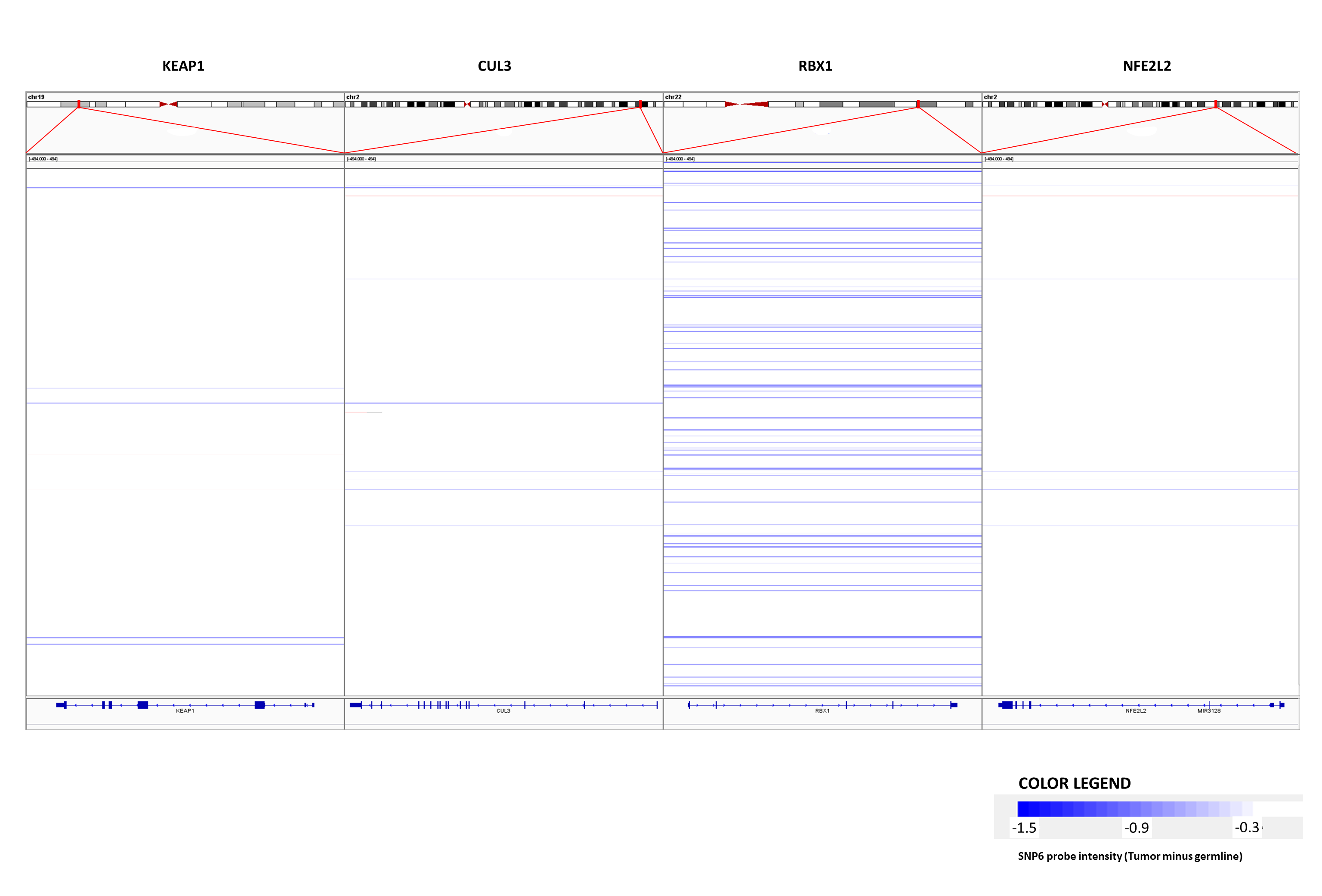

Supplement: Additional file 2: Figure S1 — DNA copy number alterations affecting components of the KEAP1/CUL3/RBX1 complex. DNA copy number affecting KEAP1/CUL3/RBX1 complex components in the TCGA PTC set. Copy number is estimated based on probe intensity derived from the Genome-Wide Human SNP Array 6.0 (Affymetrix). Around 17% of samples exhibit copy number losses (in blue, see Figure legend) affecting RBX1 gene. This appears to be a particular mechanism for gene disruption affecting this complex component, since the rest of complex components exhibit alterations at copy number level in a considerably lower frequency of samples. [file 1476-4598-12-124-S2.tiff]
